# Deoxycytidine production by a metabolically engineered *Escherichia coli* strain

**DOI:** 10.1186/s12934-015-0291-8

**Published:** 2015-07-07

**Authors:** Jin-Sook Kim, Bong-Seong Koo, Hyung-Hwan Hyun, Hyeon-Cheol Lee

**Affiliations:** ForBioKorea Co., Ltd., Siheung Industrial Center 22-321, Seoul, 153-701 Republic of Korea; Department of Bioscience and Biotechnology, Hankuk University of Foreign Studies, San 89, Wangsan-Ri, Mohyun-Myun, Yongin-Shi, 449-791 Republic of Korea

**Keywords:** Deoxycytidine, Production, Deoxynucleoside, Pyrimidine, Metabolic engineering

## Abstract

**Background:**

Rational engineering studies for deoxycytidine production were initiated due to low intracellular levels and tight regulation. To achieve high-level production of deoxycytidine, a useful precursor of decitabine, genes related to feed-back inhibition as well as the biosynthetic pathway were engineered. Additionally, we predicted the impact of individual gene expression levels on a complex metabolic network by microarray analysis. Based on these findings, we demonstrated rational metabolic engineering strategies capable of producing deoxycytidine.

**Results:**

To prepare the deoxycytidine producing strain, we first deleted 3 degradation enzymes in the salvage pathway (*deoA*, *udp*, and *deoD*) and 4 enzymes involved in the branching pathway (*dcd*, *cdd*, *codA* and *thyA*) to completely eliminate degradation of deoxycytidine. Second, *purR*, *pepA* and *argR* were knocked out to prevent feedback inhibition of CarAB. Third, to enhance influx to deoxycytidine, we investigated combinatorial expression of *pyrG*, T4 *nrdCAB* and *yfbR*. The best strain carried pETGY (*pyrG*-*yfbR*) from the possible combinatorial plasmids. The resulting strain showed high deoxycytidine yield (650 mg/L) but co-produced byproducts. To further improve deoxycytidine yield and reduce byproduct formation, *pgi* was disrupted to generate a sufficient supply of NADPH and ribose. Overall, in shake-flask cultures, the resulting strain produced 967 mg/L of dCyd with decreased byproducts.

**Conclusions:**

We demonstrated that deoxycytidine could be readily achieved by recombineering with biosynthetic genes and regulatory genes, which appeared to enhance the supply of precursors for synthesis of carbamoyl phosphate, based on transcriptome analysis. In addition, we showed that carbon flux rerouting, by disrupting *pgi*, efficiently improved deoxycytidine yield and decreased byproduct content.

**Electronic supplementary material:**

The online version of this article (doi:10.1186/s12934-015-0291-8) contains supplementary material, which is available to authorized users.

## Background

Deoxycytidine (dCyd) is a commercially useful precursor in the chemical synthesis of various drugs including decitabine (Dacogen™, 5-aza-2′-deoxycytidine), which is used to treat myelodysplastic syndromes, a class of conditions where certain blood cells are dysfunctional, and for acute myeloid leukemia [1, 2]. A similar analogue, azacitidine (5-aza-2′cytidine) derived from cytidine (Cyd) is also used for this purpose [3]. However, because azacitidine is a potential substrate for the DNA replication machinery after metabolism to its deoxy derivative, there are subtle differences in efficacy between azacitidine and decitabine. A recent report described the production of Cyd by rationally engineered *Escherichiacoli*, in which pentose phosphate pathway (PPP) genes were amplified to supply precursor [4]. And Zhu et al. also reported the production of Cyd by deregulation of the *pyr* operon and the overexpression of the *prs*, *pyrG* and *pyrH* genes in *Bacillussubtilis* [5]. In contrast, so far, the studies for dCyd production have not been achieved by rational engineering, due to low intracellular levels and tight gene regulation. To date, dCyd production has only been achieved by traditional engineering employing bacteria belonging to the genera *Corynebacterium* [6]. However, while dCyd production by rational engineering is not well studied, another pyrimidine deoxynucleoside, thymidine, has been studied actively [7–11]. In previous work, we showed that deletion of three repressors (*purR*, *pepA* and *argR*) involved in the regulation of carbamoyl phosphate synthetase (*carA*/*carB*), positively affected thymidine production [7]. To enhance the reduction of nucleotides, we overexpressed T4 NDP reductase subunits. In an effort to develop a new deoxy pyrimidine nucleoside-producing strain that might spawn similar engineering towards development of a thymidine producer, we focused on dCyd production by *E. coli.*

**Figure 1:**
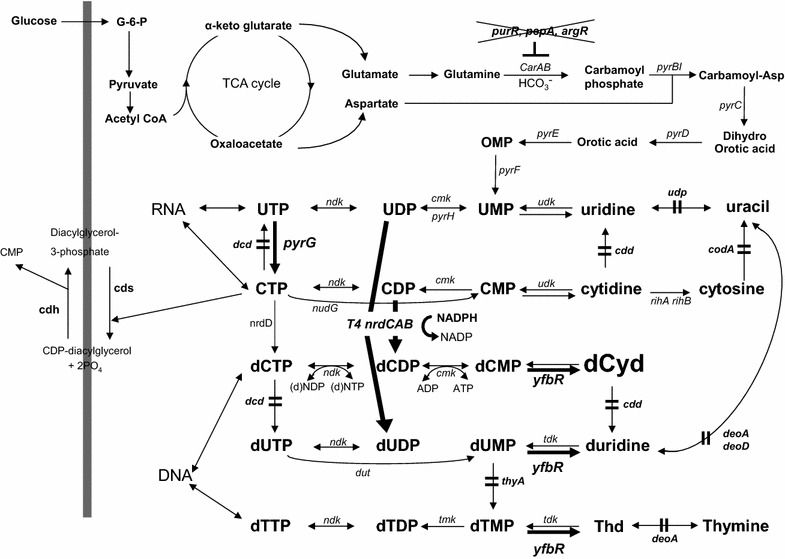
The deoxycytidine biosynthetic pathway. The steps engineered in this study are indicated by the *bold arrows* and *lines*. Components of the catabolic pathways are as follows: *carAB* carbamoyl phosphate synthase, *pyrBI* aspartate-carbamoyl transferase, *pyrC* dihydroorotase, *pyrD* dihydroorotate oxidase, *pyrE* orotate phosphoribosyl transferase, *pyrF* OMP decarboxylase, *pyrG* CTP synthetase, *pyrH* UMP kinase, *yfbR* dCMP phosphohydrolase, *nrdCAB* nucleotide diphosphate reductase, *thyA* thymidylate synthase, *dcd* dCTP deaminase, *udk* uridine kinase, *deoA* thymidine phosphorylase, *tdk* thymidine kinase, *udp* uridine phosphorylase, *dut* deoxyribonucleotide triphosphatase, *ndk* nucleotide diphosphate kinase, *tmk* TMP kinase, *cdd* cytidine deaminase, *codA* cytosine deaminase, *deoD* purine nucleoside phosphorylase, *rihA*, *rihB* ribosyl pyrimidine nucleosidase, *cmk* cytidylate kinase, *purR* purine repressor, *pepA* aminopeptidase A, *argR* arginine repressor.

As illustrated in Figure 1, de novo biosynthesis of dUMP occurs through two distinct pathways in pyrimidine biosynthesis [12, 13]. The quantitatively more important pathway involves the deamination of dCTP to dUTP by dCTP deaminase, followed by the hydrolysis of dUTP by dUTP nucleotidohydrolase (dUTPase) to yield dUMP with 75% of endogenous dUMP arising through this route. The second pathway generating the remaining 25% of dUMP consists of the reduction of UDP by ribonucleoside diphosphate reductase to dUDP, which is phosphorylated by nucleoside diphosphokinase (encoded by *ndk*) to dUTP and subsequently hydrolyzed to dUMP by dUTPase [14]. Alternatively, dUMP may be produced by pyrimidine salvage through reaction of deoxyuridine with thymidine kinase. The deoxyuridine, in turn, may arise either from dCyd through deamination catalyzed by Cyd (dCyd) deaminase (encoded by *cdd*) or by the condensation of uracil and deoxyribose 1-phosphoate mediated by thymidine phosphorylase (encoded by *deoA*), although this latter reaction is believed to act predominantly in the catabolic direction [13, 15]. As explained here, most of the dCTP biosynthetic pathway has shared pathways with dTTP biosynthesis, but dCTP is synthesized via a more complex route than dTTP.

In this study, CTP synthetase (encoded by *pyrG*) and T4 NDP reductase (encoded by *nrdCAB*) were overexpressed to increase dCMP pools. While a highly specific dCMP phosphohydrolase was needed to generate dCyd from increased dCMP, all 5′-nucleotidases studied so far are enzymes with broad specificity on NMP and dNMP (e.g. those encoded by *ushA*, *surE*, *yjjG* and *yfbR*) [16]. Especially, UshA and SurE are active on only NMP, with no apparent activity on dNMP. YjjG, known as the house-cleaning enzyme, catalyzes CMP, dUMP and dTMP as substrates but not dCMP [17]. In contrast, YfbR uses dCMP as a substrate but prefers other dNMPs, exhibiting higher activity on dGMP, dUMP and dAMP than dCMP [18]. In particular, the activity on dUMP of *E. coli yfbR* is higher than dCMP by 1.5-fold (dGMP > dUMP > dAMP > dCMP > GMP > dTMP > NMPs) [19]. Other than purine nucleotides, the higher activity of YfbR on dUMP may be problematic, because dUMP can be increased proportionally with dCMP. Among 5′-nucleotidases studied so far, however, YfbR is the best available candidate, as, to date, no highly specific dCMP phosphohydrolase has been identified or engineered.

Based on the information known about nucleotide biosynthesis and our experience developing thymidine producing strains, we prepared a novel dCyd-producing strain by rational metabolic reprogramming. Our approach, first, involved the deletion of known nucleoside degrading enzymes and branching enzymes, followed by the deletion of the thymidylate synthase gene (*thyA*). Next, to enhance influx to pyrimidine, the genes encoding the CarAB repressors were deleted and the bottleneck enzymes (NDP reductase, CTP synthetase and dCMP phosphohydrolase) were overexpressed. In this approach, unwanted problems may arise from increasing unexpected byproducts and losing control of the biosynthesis of purine, pyrimidine and arginine, which may affect cell growth and maintenance. Because purine and pyrimidine nucleotides constitute components of nucleic acids, cofactors in enzymatic reactions, intracellular and extracellular signals, phosphate donors, and the major carriers of cellular energy, imbalances between these different nucleotide pools can significantly perturb normal cellular function [20–22]. Here, we analyzed metabolic change by partial transcriptome microarray and investigated byproduct profiles of the strain derivatives generated at each engineering stage. Based on these findings, we demonstrated the use of rational metabolic engineering of *E*. *coli* to produce dCyd with less byproducts formation.

## Results

### The deletion of salvage and branching pathway genes in cells with high dCyd resistance

**Figure 2:**
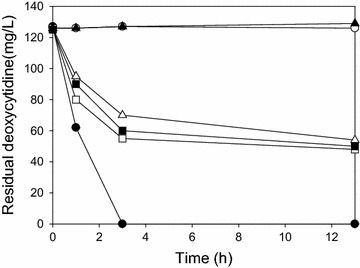
Deoxycytidine degradation assay. To eliminate possible degradation of dCyd which was produced during fermentation, known pyrimidine nucleoside degradation enzymes were disrupted. The disruption of *deoA*, *udp* and *deoD*, which are active on deoxyuridine and thymidine, was not sufficient to block dCyd degradation completely. However, after the disruption of both *dcd* and *cdd*, dCyd degradation was not observed in the in vitro assay. Negative control with dCyd (*open circle*), BL21(DE3) (*filled circle*), HLC001 (*ΔdeoA*) (*open square*), HLC003 (*ΔdeoA ΔdeoD Δudp*) (*filled square*), HLC004 (*ΔdeoA ΔdeoD Δudp Δdcd*) (*open triangle*) and HLC005 (*ΔdeoA ΔdeoD Δudp Δdcd Δcdd*) (*filled triangle*).

*E. coli* DeoA, Udp and DeoD have essential roles in pyrimidine nucleotide salvage pathways and are known to catalyze reversible reactions [9, 12]. Hence, these nucleoside phosphatases might have potential roles in the degradation of intracellular dCyd. Using a high concentration of dCyd resistant strain (up to 10 g/L, Additional file 1: Figure S1), *deoA, udp* and *deoD* were deleted sequentially by PCR-mediated disruption to construct HLC003 (Additional file 1: Figure S2A). If overall phosphatase activity of any unidentified pathway members is less than the contribution of these three enzymes (DeoA, Udp and DeoD) to the salvage pathway, the deletion of *deoA, udp* and *deoD* should be enough to prevent degradation of dCyd in at least low dCyd-producing cells. To test this notion, an in vitro dCyd degradation assay was carried out for evaluation. The assay profile, however, showed that, during the time tested, dCyd degradation in vitro was apparently not completely blocked by disrupting *deoA, udp* and *deoD* (Figure 2), perhaps because dCyd degradation is closely linked to the conversion steps of dCTP, dCyd and cytosine into dUMP, deoxyuridine and uracil, respectively [12]. Hence, this suggested the branching nodes into other nucleotide pathways would be good targets for further strain engineering to increase dCyd influx by blocking additional dCyd degradation. Based on the nucleotide synthetic pathway, *dcd*, *cdd* and *codA*, which encode enzymes that catalyze deamination of dCTP, dCyd and cytosine, respectively, were disrupted step-by-step, resulting in HLC006. Additionally, *thyA,* which plays a key role in dTTP synthesis, was disrupted in the parental HLC006 strain (Additional file 1: Figure S2B). However, because the resulting strain, HLC007, had no way to synthesize dTTP due to deletions of *thyA,deoA, deoD* and *udp*, it could not synthesize dTTP by *de novo* nucleotide synthetic pathway. Afterward, 20 mg/L of thymidine was added to all media for complementation of growth. Interestingly, in dCyd degradation assay, dCyd degradation was still observed in HLC004 strain but not in HLC005 strain (Figure 2; Table 1). This result means that the possible paths, whereby dCyd can be degraded by detouring dCyd to deoxyuridine, were blocked completely by disrupting *cdd* as well as *dcd*.

**Table 1:** Strains and plasmids

Strain or plasmid	Description^a^	Source or reference
Strains		
BL21(DE3)	F^−^ *ompT hsdS*_*B*_ *(r*_*B*_^−^ *m*_*B*_^−^ *) gal dcm me131* (DE3)	Invitrogen
XL1-Blue	*recA1 endA1 gyrA96 thi*-*1 hsdR17 supE44 relA1 lac* [F′ *proAB lacI* ^*q*^ *ZΔM15 Tn10* (Tet^r^)]	Stratagene
HLC001	BL21 *ΔdeoA*	This study
HLC003	BL21 *ΔdeoA Δudp ΔdeoD*	This study
HLC004	BL21 *ΔdeoA Δudp ΔdeoD Δdcd*	This study
HLC005	BL21 *ΔdeoA Δudp ΔdeoD Δdcd Δcdd*	This study
HLC006	BL21 *ΔdeoA Δudp ΔdeoD Δdcd Δcdd ΔcodA*	This study
HLC007	BL21 *ΔdeoA Δudp ΔdeoD Δdcd Δcdd ΔcodA* *ΔthyA*	This study
HLC010	BL21 *ΔdeoA Δudp ΔdeoD Δdcd Δcdd ΔcodA* *ΔthyA* *ΔpurR ΔpepA ΔargR*	This study
HLC015	BL21 *ΔdeoA Δudp ΔdeoD Δdcd Δcdd ΔcodA* *ΔthyA* *ΔpurR ΔpepA ΔargR* *Δpgi*	This study
Plasmids		
pETDuet	*ColE1* replicon, *bla*	Novagen
pKD3	Template plasmid, derivative of pANTSγ, FRT-flanked *cat*	[43]
pKD20	λ Red helper plasmid, derivative of pINT-ts, *araC*-*P*_*araB*_ and γ β exo DNA fragments	[43]
pCP20	*bla* and *cat*, *ori* ^TS^, thermal inducible FRT recombinase	[43]
pETNG	pETDuet T4 *nrdCAB* operon and *pyrG* expression under *tac* promoter, *bla*	This study
pETNY	pETDuet T4 *nrdCAB* operon and *yfbR* expression under *tac* promoter, *bla*	This study
pETGY	pETDuet *pyrG* operon and *yfbR* expression under *tac* promoter, *bla*	This study
pETNGY	pETDuet T4 *nrdCAB* operon, *pyrG* and *yfbR* expression under *tac* promoter, *bla*	This study

^a^Gene Accession: *deoA* (CAQ34741)*, udp* (CAQ34184)*, deoD* (CAQ34743)*, purR* (CAQ32135), *pepA* (CAQ34607)*, argR* (CAQ33565)*, dcd* (CAQ32477), *cdd* (CAQ32548)*, codA* (CAQ30812)*, thyA* (CAQ33153)*, pgi* (CAQ34374)*, yfbR* (CAQ32693)*, pyrG* (CAQ33104)*, T4 nrdA* (NP_049845)*, T4 nrdB* (NP_049841), *T4 nrdC* (NP_049698).

### The elimination of CarAB repressors increases the influx of precursors into nucleotide synthesis

**Figure 3:**
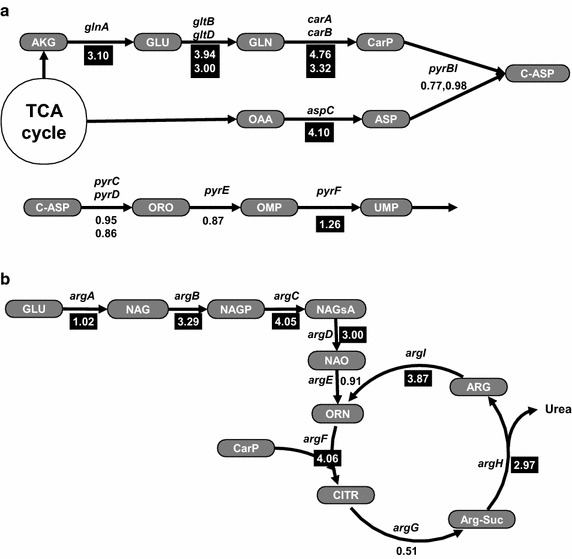
Transcriptional change of pyrimidine and arginine biosynthetic genes by disruption of CarAB repressors (PurR, PepA and ArgR). The numbers are the ratios of the expression levels in HLC010 vs. HLC007. The *shaded numbers* indicate significantly up-regulated genes in HLC010 (P < 0.05). **a** The transcriptional change of genes for precursor supply and *pyr* operon. α-ketoglutarate and oxaloacetate introduced from the TCA cycle might be converted quickly into carbamoyl phosphate and aspartate. The resulting increased precursors can enhance the production of dCyd. **b** The transcriptional change of the *arg* operon. Most of the Arg biosynthetic genes were up-regulated except for *argG*. The decrease of *argG* transcription may be the rate-limiting step for synthesizing arginine. *AKG* α-ketoglutarate, *OAA* oxaloacetate, *GLU* l-glutamate, *GLN* l-glutamine, *CarP* carbamoyl phosphate, *ASP* l-aspartate, *C-ASP* carbamoyl aspartate, *ORO* orotate, *OMP* orotate mono phosphate, *NAG* *N*-acetyl glutamate, *NAGP* *N*-acetyl glutamate phosphate, *NAGsA* *N*-acetyl glutamate semi aldehyde, *NAO* *N*-acetyl ornithine, *ORN* ornithine, *CITR* citrulline, *Arg-Suc* argino succinate, *ARG* l-arginine.

**Figure 4:**
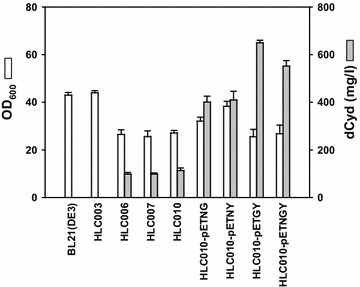
dCyd production by all engineered BL21 derivative strains. Seed cultures were grown in 5 mL of Luria–Bertani media overnight, and then transferred to 500-mL baffled flasks containing 50 mL of production media (see “Methods”). The strains carrying expression plasmids were induced for protein expression with isopropyl β-d-1-thiogalactopyranoside (IPTG; 0.5 mM) when cells reached OD_600_ ≈ 0.6. All flask cultures were incubated at 37°C and 250 rpm for 36 h. The dCyd concentration in the media was determined from the supernatants of cultivation samples. Values are mean ± SD of triplicate flasks experiments performed at the same time.

*E. coli* CarAB is controlled tightly by at least five transcription factors. Among known transcription factors, PurR, PepA and ArgR are capable of controlling CarAB activity by the coordination of intracellular levels of arginine and nucleotides [21]. We previously demonstrated that the disruption of three repressors governing *carAB* transcription could be leveraged to enhance thymidine production in *E. coli* [7]. Here, we speculated that the disruption of three repressors governing *carAB* transcription could be similarly applied to dCyd production. Using HLC007, the repressor genes, *purR, pepA* and *argR* were deleted sequentially by the same deletion method, resulting in HLC010 (Additional file 1: Figure S2C). We hypothesized that CarAB activity in HLC010 might scarcely be affected by intracellular nucleotide or nucleoside levels, and subsequently, influx of nucleotide precursors could be increased by less regulation. To test this hypothesis, we analyzed transcriptional levels of genes related to the supply of precursors by microarray assay. Not surprisingly, the transcription levels of tested genes, which synthesize carbamoyl phosphate and aspartate, were increased significantly (P < 0.05) (Additional file 1: Table S1), when PurR, PepA and ArgR were absent (Figure 3, black colored box), indicating that the increase in the supply of major precursors was caused by enhanced transcriptional levels of the tested genes. These results could not completely explain these phenomena, however, because we only tested a limited number of genes without measuring intracellular nucleoside and nucleotide levels. Regardless, this result showed that the transcription of *carAB* as well as many genes involved in supplying precursors was increased significantly under the same conditions, and subsequently dCyd yield was also increased by 1.2 fold (Figure 4). Taken together, these results supported our hypothesis.

### The overexpression of nucleotide biosynthetic genes favors dCyd production

The HLC010 strain was potentially a good host for producing dCyd because most of its intrinsic genes related to degradation of dCyd and regulation of CarAB were deleted. Accordingly, if proper nucleotide biosynthetic genes were overexpressed in this host cell, it might be a good dCyd producing strain. Considering the overall nucleotide biosynthetic pathway (Figure 1), we selected three essential steps and their corresponding genes, NDP reduction (*nrdCAB*), UTP amination (*pyrG*), and dCMP hydrolysis (*yfbR*). The overexpression of NDP reductase (NrdCAB) is known as the best way to increase intracellular dNTP level [9]. Also, as the transcription of the *pyrG* gene is regulated by a CTP pool, the overexpression of CTP synthetase (PyrG) can overcome the limitation of the excessive synthesis of CTP. The overexpression of cytidine phosphohydrolase (YfbR) can result in accelerating dCyd synthesis by the demand of dCMP for DNA synthesis. In case of NDP reductase, we used T4 NDP reductase which was previously used successfully for thymidine production [9], instead of *E. coli* intrinsic enzyme, to eliminate possible feedback inhibition by the nucleotide pool.

To test their combinatorial effect on dCyd production, T4 *nrdCAB* + *pyrG*, T4 *nrdCAB* + *yfbR*, *yfbR* + *pyrG*, and *yfbR* + *pyrG* + T4 *nrdCAB* were each assembled into synthetic operons under the control of a Tac promoter in plasmid, pETDuet. The resulting plasmids, pETNG, pETNY pETGY, and pETNGY permitted combinatorial co-expression of T4 *nrdCAB*, *yfbR* and *pyrG* (Additional file 1: Figure S3). Following expression of each of the synthetic combinatorial operons in HLC010 cells (Additional file 1: Figure S4), we investigated dCyd production in flask cultures. In HLC010-pETNG, which overexpressed T4 *nrdCAB* and *pyrG*, 401 mg/L of dCyd were produced in flask culture (Figure 4, Additional file 1: Figure S5). This dCyd yield was approximately 4-fold greater, compared to host cells, HLC010. In addition, HLC010-pETNY, which overexpressed T4 *nrdCAB* and *yfbR*, produced 410 mg/L of dCyd. These results indicated that the overexpression of two genes with distinct roles (*nrdCAB* and *yfbR*), was more effective than overexpression of similar genes related to the supply of cytidine nucleotide (*nrdCAB* and *pyrG*). Before testing HLC010-pETGY, we investigated dCyd production by HLC010-pETNGY, which appeared to be the best strain. In case of HLC010-pETNGY, which overexpressed T4 *nrdCAB*, *yfbR* and *pyrG*, dCyd yield was highly increased to 552 mg/L, as expected. However, as dCyd production was increased, several byproducts were also increased (Additional file 1: Figure S5F). In contrast, in case of HLC010-pETGY, which overexpressed only *yfbR* and *pyrG*, the quantity of byproducts was decreased in the HPLC profile, compared to HLC010-pETNGY. In return, dCyd production yield of HLC010-pETGY was increased to 650 mg/L, with less byproduct formation (Figure 4, Additional file 1: Figure S5G). In HLC010-pETGY, most of the distinct peaks were decreased in the HPLC profile, compared to HLC010-pETNGY.

### The yield of dCyd can be improved by disrupting *pgi* in HLC010-pETGY

**Figure 5:**
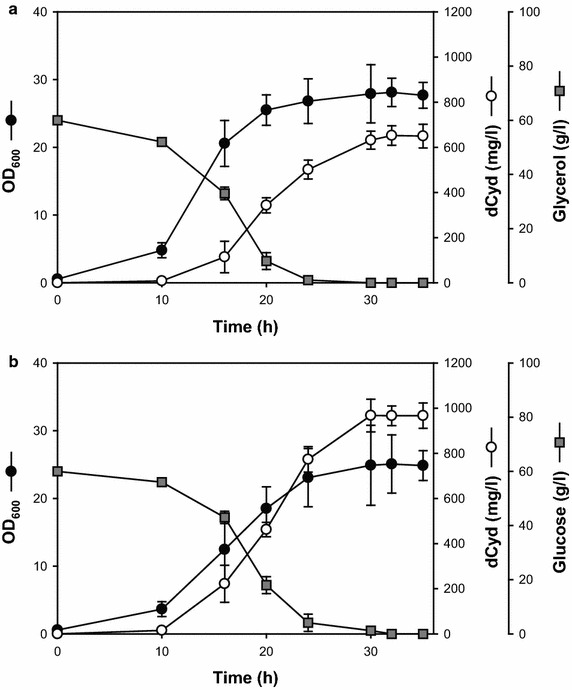
Cultivation profiles of HLC010-pETGY and HLC015-pETGY in flask scale. HLC015-pETGY showed slower growth rate but higher productivity than HLC010-pETGY by additional *pgi* deletion. **a** Fermentation profile using HLC010-pETGY. Glycerol was used as the carbon source. **b** Fermentation profile using HLC015-pETGY. Glucose, at a concentration (60 g/L) equivalent to that of cultures with glycerol, was used as the carbon source. Values are mean ± SD from three independent experiments performed in triplicate.

Eliminating *pgi*, a gene necessary for the EMP pathway, completely blocks isomerization of glucose 6-phosphate to fructose 6-phosphate, detouring the metabolism of glucose to the pentose phosphate (PP) pathway. Since the main role of the PP pathway is to supply NADPH and ribose, shifting metabolism towards this pathway could increase NADPH levels and ribose precursor in cells [7]. The disruption of *pgi* in HLC010 was performed using a linear PCR fragment with 50 nucleotides of homology extensions to construct strain HLC015 (Additional file 1: Figure S3D). The best plasmid, pETGY, as determined above, was used to transform HLC015 cells. In this experiment, we needed to change the carbon source in the medium from glycerol to glucose, because HLC015-pETGY cannot use glycerol efficiently via the PP pathway. HLC015-pETGY did not grow in production medium with glycerol, and likewise, HLC010-pETGY showed decreased growth in production medium with glucose. Accordingly, to compare these two strains, we used the optimal cultivation medium for each. Under these conditions, HLC010-pETGY produced 650 mg/L of dCyd in the production medium with glycerol, and HLC015-pETGY produced 967 mg/L of dCyd in the production medium with glucose during similar cultivation times (30 and 32 h, respectively) (Figure 5). Generally, *pgi* disruption prevents or delays the normal utilization of glucose in cells, which retards cell growth due to a limited capacity for the re-oxidation of overproduced NADPH [23, 24]. However, in HLC015-pETGY, the retardation of cell growth was not observed, suggesting that the over-accumulation of NADPH and ribose precursors was alleviated due to NADPH-recycling and ribose consumption during dCyd overproduction. Interestingly, the byproduct peaks also disappeared or considerably decreased in HPLC profiles of the broth supernatant (Additional file 1: Figure S5H). In return, the dCyd production yield of HLC015-pETGY was considerably increased, which could also be seen as increased yield caused by decreasing the byproducts. One of our objectives was to prepare a high dCyd producer with no byproducts like deoxyuridine, cytosine, uracil or thymine, while retaining the ability to produce dCyd. Even though the optimization of overall fermentation processes is still required, dCyd production was achieved successfully without significant co-produced byproducts, thereby demonstrating the potential of our engineered cells for developing industrial dCyd production and purification processes.

## Discussion

In this study, we developed promising dCyd producing strains by metabolic reprogramming of *E*. *coli*, based on recent information on pyrimidine metabolism and uncovered the metabolic change in our cells by transcriptome analysis. Specifically, the remarkable feature of our strain is that the approach produced the reduced form of cytidine by controlling regulations as well as pathway recombineering. Indeed, dCyd production by rational design was relatively more difficult than the oxidized form in terms of reducing power regeneration as well as supply of precursors. In this study, to complement a reducing power in all strains but HLC015-pETGY, glycerol was used as carbon source instead of glucose. We were supposed that HLC015-pETGY also had higher NADPH/NADP ratio than other strains, because glucose can be passed through PP pathway with generating NADPH, but glycerol cannot. In similar case of previous study for developing thymidine producer, we have obtained the supporting data that NADPH/NADP ratio of *pgi*-disrupting cells showed higher level in continuous culture with glucose, while NADH/NAD ratio showed lower, compared to those of parental cells in continuous culture with glycerol [11]. The fermentation using glycerol has an advantage of producing extra NADH, which can be easily converted into NADPH in the presence of extra phosphate [13, 25, 26]. In addition, its effect can be amplified by overexpressing intrinsic NAD kinase or soluble transhydrogenase [10, 11, 27, 28]. While there are less supporting data as to which approach is more efficient between PP pathway rerouting and glycerol fermentation, both approaches highlight the importance of supplying reducing power.

Interestingly, HPLC analysis of the culture supernatants of HLC010-pETNGY and HLC010-pETGY broth revealed the formation of byproducts in addition to dCyd production (Additional file 1: Figure S5), which potentially lowers dCyd productivity and makes downstream purification processes difficult. HLC010-pETGY showed higher productivity, compared to HLC010-pETNGY, and co-produced less byproducts (Figure 4). One possible explanation is that the excessive reduction of UDP by strong overexpression of T4 NDP reductase might have a negative effect on UTP amination by PyrG. In the case of HLC010-pETNGY, NDP reductase was overexpressed without any regulation, probably acting to convert more UDP into dUDP, while simultaneously decreasing the UTP supply for conversion to CTP by PyrG. Subsequently, excess dUMP can be readily degraded into deoxyuridine by YfbR, which has higher activity on dUMP by twofold compared to dCMP [19], because there is no pathway to convert deoxyuridine and dUTP into deoxycytidine and dCTP, respectively, in HLC010 cells where *dcd*, *cdd* and *codA* were deleted. In contrast, in HLC010-pETGY cells, more UTP can be converted into CTP by the expressed PyrG than in HLC010-pETNGY cells. Thus, if we express a dCMP phosphohydrolase with higher affinity for dCMP than *yfbR*, the simultaneous expression of a NDP reductase might be effective to improve productivity.

In HLC015-pETGY, more NADPH and ribose precursors were supplied by rerouting from the EMP pathway to the PP pathway, compared to HLC010-pETGY. The PyrG-associated supply of sufficient precursors and redox potential could have given rise to the increased dCMP/dUMP ratio of HLC015-pETGY cells, leading to enhanced dCMP hydrolysis by YfbR. In support of this, we obtained several lines of evidence that byproduct formation was further decreased when YfbR was expressed under control of the T7 promoter, compared to the arabinose promoter (data not shown). While further experiments are needed to clearly explain the relationship of YfbR activity and dCyd biosynthesis, we postulate that the balance between YfbR and PyrG activity may be very closely linked to byproduct formation in HLC010-pETGY.

In further engineering, the complete elimination of degradation enzymes with broad activity may be very important in further developing a dCyd producer, because dCyd is released to the media, instead of accumulating inside cells. While not investigated here, it is possible that ribonucleoside hydrolases and analogues, RihA, RihB, and RihC may be involved in dCyd nicking via an unknown mechanism [29]. This would explain how residual nucleoside byproducts still existed in vivo, even though the in vitro dCyd degradation assay showed HLC005 appeared to block dCyd hydrolysis completely. The contribution of RihA, B, and C for byproduct formation remains to be examined in vivo. Nonetheless, this potential activity of RihA, B, and C is not entirely unexpected in light of the other degradation enzymes for dCyd (or Cyd). For example, a pyrimidine requiring *cdd* mutant of *E. coli* can utilize cytidine as a pyrimidine source by an alternative pathway, which involves hydrolysis of cytidine by RihA, B, and C [29]. Likewise, RihA, B, and C are predicted to fulfill degradation roles for dCyd/Cyd in our cells by participating in the salvage pathway.

As mentioned, CarAB is controlled by at least five transcription factors [30, 31]. Among these repressors, those encoded by *purR*, *pepA*, and *argR* are known as representative binders to the *carAB* promoter P1 (upstream) and P2 (downstream), which are adjacent to each other [32]. PurR and ArgR repress transcription by binding to each *carAB* promoter P1 and P2, respectively. In contrast, PepA shares a binding site with PurR and ArgR partially, so it influences regulation of both promoters. According to previous study, in the case of purine metabolism in *purR*-deficient cells, genes related to ribose moiety supply and the flux into IMP, are generally increased but genes related to AMP or GMP biosynthesis from IMP are decreased in expression [21]. By eliminating *argR*, many genes in arginine and aspartate biosynthesis, which are directly regulated by the ArgR repressor, are up-regulated [33–35]. Likewise, the third repressor PepA influences both metabolism and the intracellular nucleotide pool. However, the simultaneous disruption of *purR*, *pepA* and *argR* may appear to give cells more complex metabolic changes in nucleotide metabolism and arginine metabolism, compared to the separate one gene disruption.

The arginine biosynthetic pathway is competitive with the pyrimidine biosynthetic pathway in utilizing carbamoyl phosphate [21, 33]. If arginine overproduction occurred in our strain, this would be disadvantageous to pyrimidine biosynthesis. However, in this study, we did not observe arginine overproduction in the three repressor-deficient cells, while dCyd overproduction was observed. Interestingly, down-regulation of ArgG in the three repressor-deficient cells was observed. One possible explanation for this is that the down-regulation of ArgG may resulted from co-disruption of *pepA*/*purR* and amplification of other nucleotide synthetic pathways in HLC010. This would explain why previous studies, which all involved *argR* regulation but not co-disruption of *pepA* and *purR*, did not observe *argG* down-regulation. Even though we cannot explain the clearly complex regulation in the recombineered cells, it is plausible that the decrease of ArgG expression in the three repressor-deficient cells slowed the conversion rate of carbamoyl phosphate into the arginine precursor, thereby enabling metabolism of carbamoyl phosphate mainly via the pyrimidine pathway.

As shown in Figure 3a, expression of *pyrBI*, *pyrC*, *pyrD* and *pyrE* were kept off or slightly down-regulated in HLC010, which is consistent with the earlier observation that these genes are kept off even in the absence of the repressors encoded by *purR*, *pepA* and *argR*, through attenuation control by coupled transcription and translation [36]. We speculate that this attenuation might be the result of increased intracellular UTP and CTP levels that arise in the metabolically engineered strain. In support of this notion, the transcription of *pyrBI* and *pyrE* are controlled negatively by UTP-sensitive attenuation [37–39]. Additionally, the transcription of *pyrBI* is regulated by reiterative transcription at high UTP levels [40]. Translation of *pyrC* is controlled negatively by preventing ribosome binding to the Shine–Dalgarno (SD) box via shifting transcription start sites at high CTP levels [41, 42]; *pyrD* may be regulated by a similar mechanism [43]. Such UTP- and CTP-sensitive regulation of these genes might limit pyrimidine biosynthesis, even when the supply of precursors and the downstream dCyd flux are increased. Despite severe UTP- and CTP-sensitive regulation, the additional overexpression of genes of the *pyr* operon in our cells may be a reliable approach.

## Conclusions

From a technological standpoint, a notable outcome of the studies described here was the preparation of a dCyd producing strain with fewer byproducts by rational metabolic engineering. We demonstrated that high dCyd yield could be readily achieved by recombineering in combination with disruption of dCyd degrading enzymes and repressors for carbamoyl phosphate synthetase, and amplification of dCyd synthesis enzymes. In addition, we showed that carbon flux rerouting by disrupting *pgi*, was a very efficient approach in our cells. In particular, we showed how nucleotide metabolic flow was changed by microarray analysis, which could easily be leveraged for further improvement of the dCyd-producing strain.

## Methods

### Bacterial strains and growth conditions

All strains used in this study are shown in Table 1. Parental strain *E. coli* BL21(DE3) was used for preparing dCyd producing strains. To eliminate the possibility of dCyd degradation by endogenous nucleosides degradation enzymes, a triple knockout strain was prepared by sequential knockout of *deoA*, *udp* and *deoD* in BL21(DE3) by PCR mediated gene disruption as described previously [44]. The first-step generated strain HLC003, which was then subject to disruption of genes encoding branching enzymes (*dcd*, *cdd*, *codA* and *thyA*), followed by removal of the chloramphenicol marker (Cm) in each target gene using pCP20 by the same method. The resulting strain, HLC007 was then subject to disruption of genes encoding the CarAB repressors (*purR*, *pepA* and *argR*), resulting in strain HLC010. Finally, the *pgi* gene of HLC010 was knocked out by the described method, resulting in strain HLC015 (BL21(DE3) *ΔdeoA Δudp ΔdeoD Δdcd Δcdd ΔcodAΔthyAΔpurR ΔpepA ΔargRΔpgi*::*Cm*). *E. coli* XL1-Blue was used for cloning genes.

Typically, cultures were grown in Luria–Bertani (LB) medium supplemented with antibiotic as needed. Antibiotics were provided at the following concentrations: chloramphenicol (Cm), 20 μg/mL; ampicillin (Amp), 100 μg/mL.

For dCyd production experiments, a suspension of cells was inoculated into a 250 mL flask containing 50 mL of LB medium and incubated at 37°C and 250 rpm for 8 h. For flask culture, 5 mL of culture broth was transferred to a 500-mL baffled flask containing 50 mL of production medium (60 g/L glycerol (glucose in case of HLC015), 10 g/L CaCO_3_, 10 g/L yeast extract, 0.4 g/L MgSO_4_·7H_2_O, 14.84 g/L soytone, 100 μg/mL ampicillin, and trace elements) and 0.5 mM IPTG for induction, and then incubated at 37°C and 250 rpm for 36 h.

### Plasmid construction

All plasmids used in this study are listed in Table 1 and all primer sequence used for constructing vectors are listed in Additional file 1: Table S2. For expression of the dCyd biosynthetic genes T4 *nrdCAB*, *pyrG* and *yfbR*, plasmid pETDuet (Novagen) was used in this study. To generate plasmids pETNG and pETNY, PCR-amplified T4 *nrdB* (P23 and P24) was cloned into the NcoI/SalI restriction sites of pETDuet, and then PCR-amplified *pyrG* and *yfbR* (P27 and P28) were cloned into the NdeI/XhoI restriction sites, respectively. PCR-amplified T4 RBS-*nrdCA* (P25 and P26) was then ligated into each of the above plasmids using the SalI restriction site (Additional file 1: Figure S3A, B). Plasmid pETGY was generated by inserting PCR-amplified *pyrG* (P29 and P30) into the NcoI/SalI restriction sites and then inserting PCR-amplified *yfbR* (P31 and P32) into the NdeI/XhoI restriction sites of pETDuet (Additional file 1: Figure S3C). To generate the plasmid pETNGY, PCR-amplified T4 *nrdB* (P23 and P24) was inserted into the NcoI/SalI restriction sites of pETDuet and then PCR-amplified *pyrG* (P27 and P28) was cloned into the NdeI/XhoI restriction sites. PCR-amplified RBS-*yfbR* (P33 and P32) was inserted into the XhoI restriction site of the resulting plasmid. After checking gene orientation, PCR-amplified T4 RBS-*nrdCA* (P25 and P26) was ligated into the SalI restriction site (Additional file 1: Figure S3B, D). All plasmids were confirmed by DNA sequencing.

### In vitro dCyd degradation assay

Equal amounts of transformed cells were harvested by centrifugation and washed with 10 mM Tris (pH 7.4) containing a protease inhibitor mixture (Complete™, Amersham Phamacia). Each enzyme solution was prepared by sonication and its protein concentration was determined by the Lowry assay. For the enzyme assay, 100 μL of 1 mM dCyd was mixed with 100 μL of enzyme solution (1.2 mg/mL) and the reaction mixture was incubated at 30°C for 16 h with sampling for analysis (1, 3, and 13 h). Then enzyme reaction was stopped by the filtration with PVDF and the residual thymidine concentration was measured in the reaction mixture.

### Transcriptome analysis

Total cellular RNA was extracted from mid-log phase cells with a QIAGEN RNEasy Mini Kit (Qiagen, Valencia, CA, USA) as described by the manufacturer. RNase-free DNaseI (Takara Bio, Shiga, Japan) was used during the isolation procedure to eliminate possible DNA contamination. cDNA probes for cDNA microarray analysis were prepared by the reverse-transcription of total RNA (25 μg) in the presence of aminoallyl-dUTP and 6 μg of random primers (Invitrogen, Carlsbad, CA) for 3 h. The cDNA probes were cleaned up using Microcon YM-30 columns (Millipore, Bedford, MA, USA) and then coupled to Cy3 dye (for reference HLC007) or Cy5 dye (for test sample HLC010) (Amersham Pharmacia, Uppsala, Sweden). The Cy3- or Cy5-labeled cDNA probes were purified with QIAquick PCR Purification Kit (Qiagen, Valencia, CA). Dried Cy3- or Cy5-labeled cDNA probes were resuspended in hybridization buffer (30% formamide, 5X SSC, 0.1% SDS, and 0.1 mg/mL salmon sperm DNA). The Cy3- or Cy5-labeled cDNA probes were mixed together and hybridized to a customized microarray slide (Mycroarray.com, Ann Arbor, MI, USA). After washing and drying slide, which was scanned by Axon 4000B (Axon Instrument, Union City, CA, USA) to generate hybridization images.

Hybridization images were analyzed by GenePix Pro 3.0 software (Axon Instrument, Union City, CA, USA) to obtain gene expression ratios (M value = (Cy5 signal_HLC010_ − background)/(Cy3 signal_HLC007_ − background)). Microarray data analysis was carried out by Genowiz 4.0™ (Ocimum Biosolutions, India). The mean signal intensity values of the duplicate spots were averaged (A value = [(Cy5 signal_HLC010_ − background) + (Cy3 signal_HLC007_ − background)]/2) and then normalized by the global normalization method.

### Analytical methods

Biomass measured by the OD_600_ was converted to DCW using a standard curve (1.0 OD_600_ = 0.45 g_dcw_/L). Quantitative determinations of bases and nucleosides were performed by HPLC with modifying previous condition for thymidine [9]. The operating conditions were as follows: column, Zorbax SB-C18, 5 μm (4.6 × 150 mm) (Agilent Co., Ltd., Santa Clara, CA, USA); solvent, 1% (v/v) acetonitrile in water; flow rate, 1 mL/min; detection at 265 nm. All standards for HPLC were purchased from Sigma.

## Additional file

**Additional file 1:** In the Supplemental Figure Section results from strain screening experiment and respective SDS-PAGE harboring each plasmid with map as well as HPLC profiles are presented. In the Supplemental Table Section the original data of transcriptome analysis and all primers used in this study are presented.
