# Supplementary material for: Deoxycytidine production by a metabolically engineered Escherichia coli strain
Source: Microb Cell Fact. 2015 Jul 7;14:98. doi: 10.1186/s12934-015-0291-8 (PMC4491880; doi:10.1186/s12934-015-0291-8)
Supplement: Supplementary file 1 — Additional file 1: In the Supplemental Figure Section results from strain screening experiment and respective SDS-PAGE harboring each plasmid with map as well as HPLC profiles are presented. In the Supplemental Table Section the original data of transcriptome analysis and all primers used in this study are presented. [file 12934_2015_291_MOESM1_ESM.pdf]

# Supplementary information File

**“Deoxycytidine production by a metabolically engineered *Escherichia coli* strain”**

Jin-Sook Kim, Bong-Seong Koo, Hyung-Hwan Hyun, Hyeon-Cheol Lee*

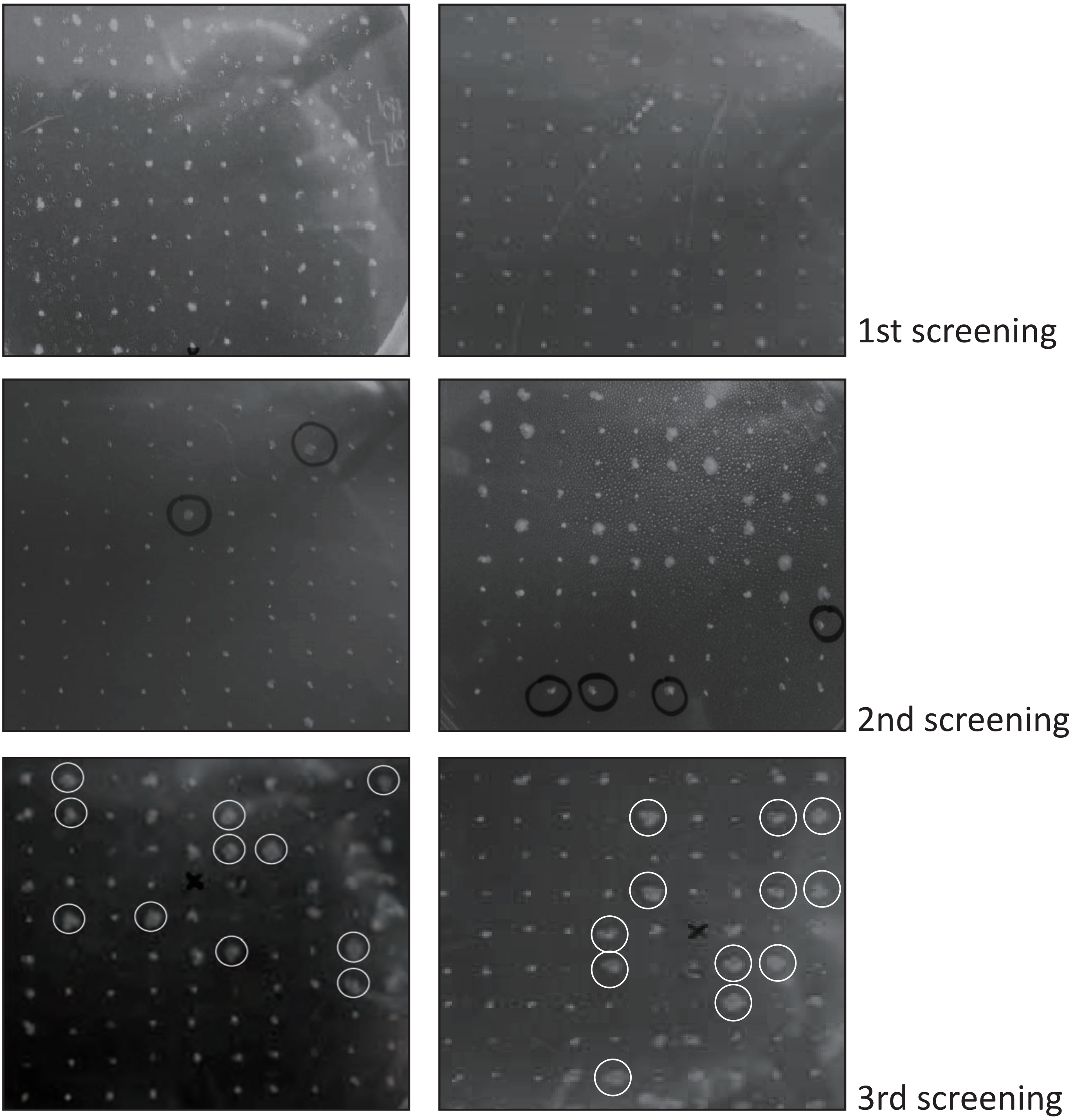


**Supplementary Fig. S1. Screening of high-concentration dCyd resistant strains.** Cells were grown on M9 minimal agar plate containing 20 g/L deoxycytidine for 1 day, which was maintained at 37℃. By repetitive rounds of selection, dCyd resistant strains were enriched.

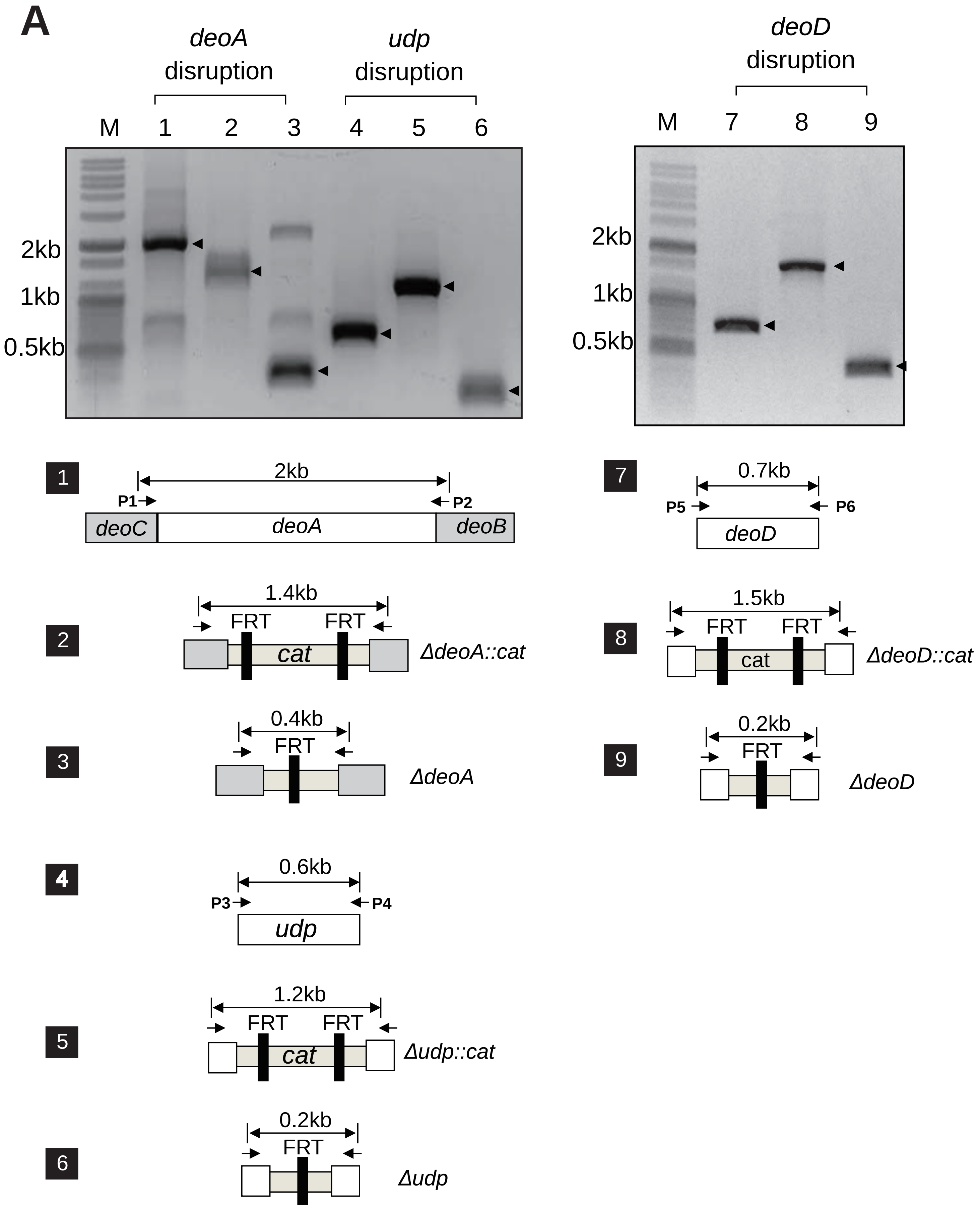


**Supplementary Fig. S2**. PCR analysis of 3 disruption mutants. P1–P22 refer to priming sites (Table S1). PCR amplification to identify gene deletion of each target gene was performed using genomic DNA prepared from each of the *E. coli* BL21 derivatives as templates. (A) Salvage pathway gene disruption. M: size marker; lane 1: *deoC* and *deoB* region including *deoA* (2.0 kb); lane 2: *ΔdeoC::cat::ΔdeoB* (1.4 kb); lane 3: *Δ(deoC-deoB)* (0.4 kb), represented to *ΔdeoA*; lane 4: *udp* (0.6 kb); lane 5: *Δudp::cat* (1.2 kb); lane 6: *Δudp* (0.2 kb); lane 7: *deoD* (0.7 kb); lane 8: *ΔdeoD::cat* (1.5 kb); lane 9: *ΔdeoD* (0.2 kb). FRT (black bar) - flanked chloramphenicol resistant gene was amplified by PCR. The linear disruption PCR fragment was transformed into λ Red recombinase expressed strain and then chloramphenicol resistant transformants were selected. The selective marker was eliminated by FLP recombinase system.

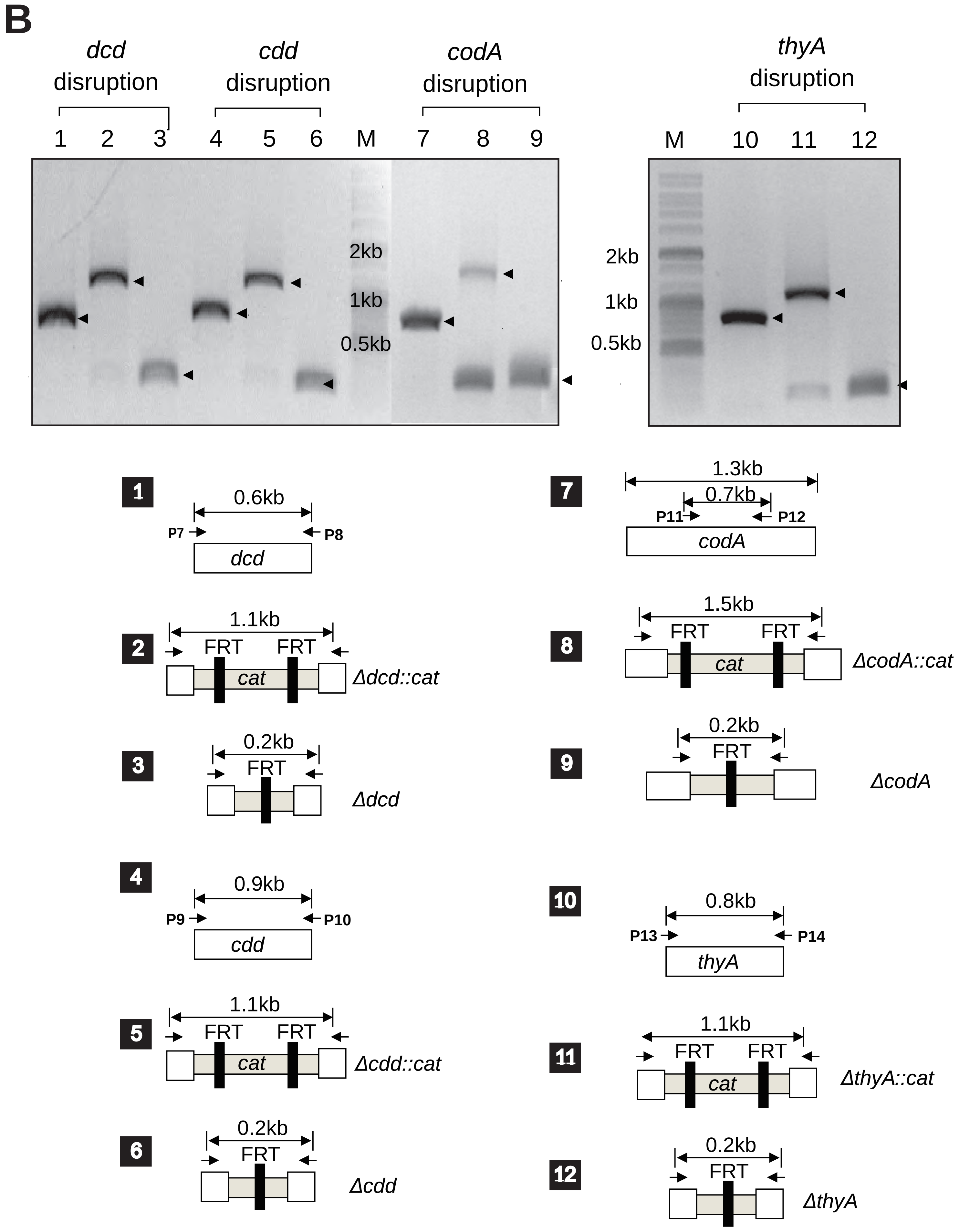


**Supplementary Fig. S2.** PCR analysis of 3 disruption mutants. P1–P22 refer to priming sites (Table S1). PCR amplification to identify gene deletion of each target gene was performed using genomic DNA prepared from each of the *E. coli* BL21 derivatives as templates. (B) ; lane 1: *dcd* (0.6 kb); lane 2: *Δdcd::cat (*1.1 kb); lane 3: *Δdcd* (0.2 kb); lane 4: *cdd* (0.9 kb); lane 5: *Δcdd::cat* (1.1 kb); lane 6: *Δcdd* (0.2 kb); lane 7: *codA* (0.7 kb); lane 8: *ΔcodA::cat* (1.5 kb); lane 9: *ΔcodA* (0.2 kb); lane 10: *thyA* (0.8 kb); lane 11: *ΔthyA::cat* (1.1 kb); lane 12: *ΔthyA* (0.2 kb).

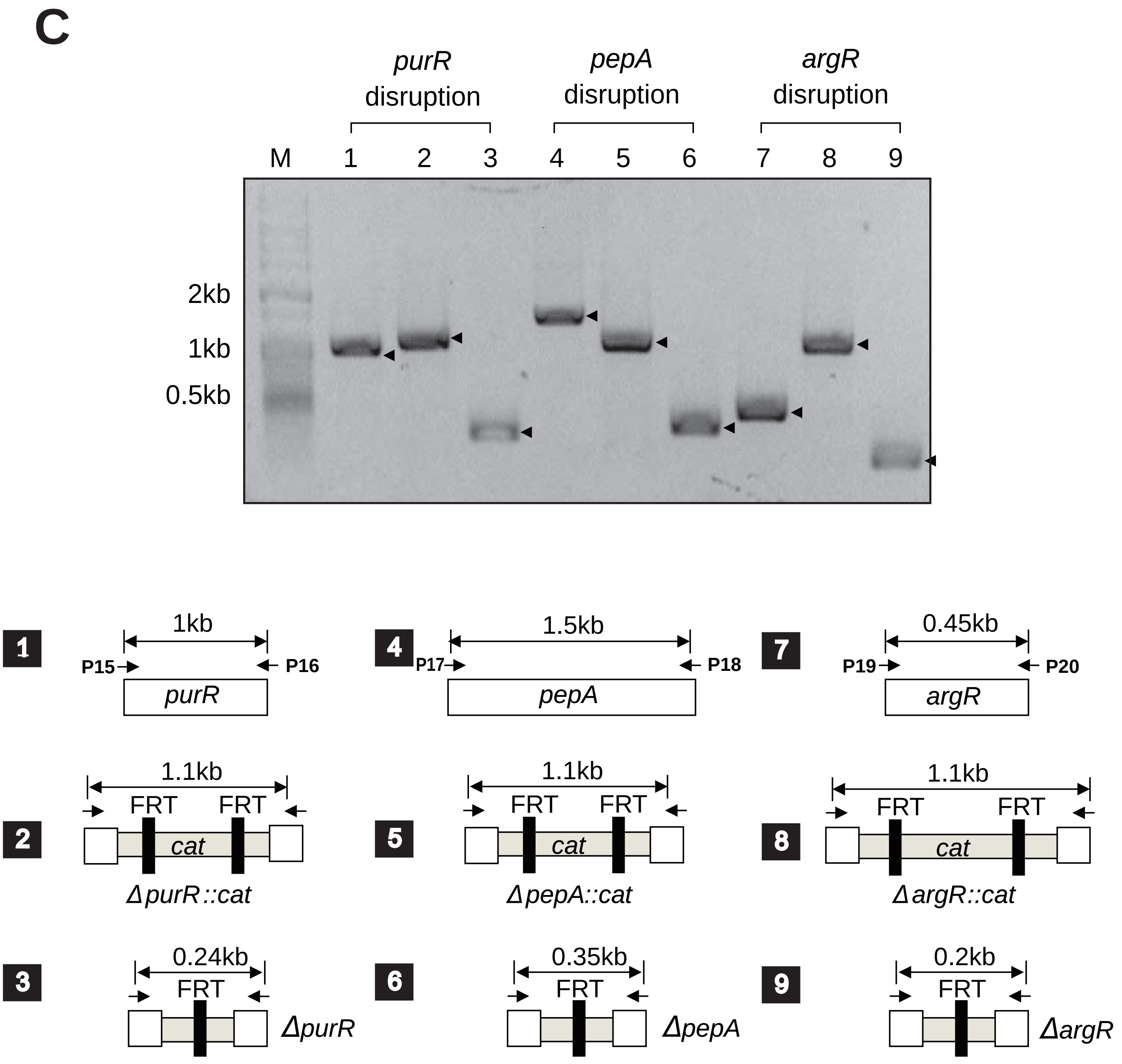


**Supplementary Fig. S2.** PCR analysis of 3 disruption mutants. P1–P22 refer to priming sites (Table S1). PCR amplification to identify gene deletion of each target gene was performed using genomic DNA prepared from each of the *E. coli* BL21 derivatives as templates. (C); lane 1: *purR* (1.0 kb); lane 2: *ΔpurR::cat* (1.1 kb); lane 3: *ΔpurR* (0.24 kb); lane 4: *pepA* (1.5 kb); lane 5: *ΔpepA::cat* (1.1 kb); lane 6: *ΔpepA* (0.35 kb); lane 7: *argR* (0.45 kb); lane 8: *ΔargR::cat* (1.1 kb); lane 9: *ΔargR* (0.2 kb).

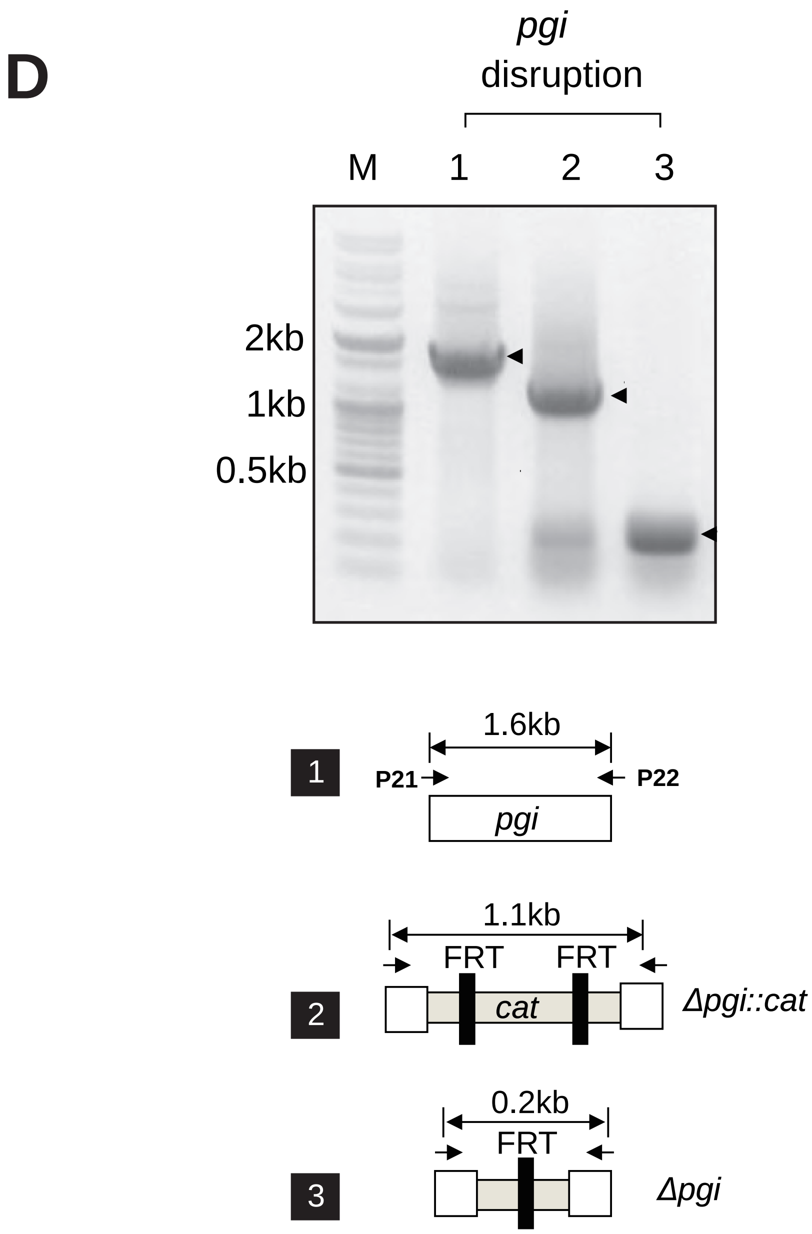


**Supplementary Fig. S2.** PCR analysis of 3 disruption mutants. P1–P22 refer to priming sites (Table S1). PCR amplification to identify gene deletion of each target gene was performed using genomic DNA prepared from each of the *E. coli* BL21 derivatives as templates. (D) lane 1: *pgi* (1.6 kb); lane 2: *Δpgi::cat* (1.1 kb); lane 3: *Δpgi* (0.2 kb).

A

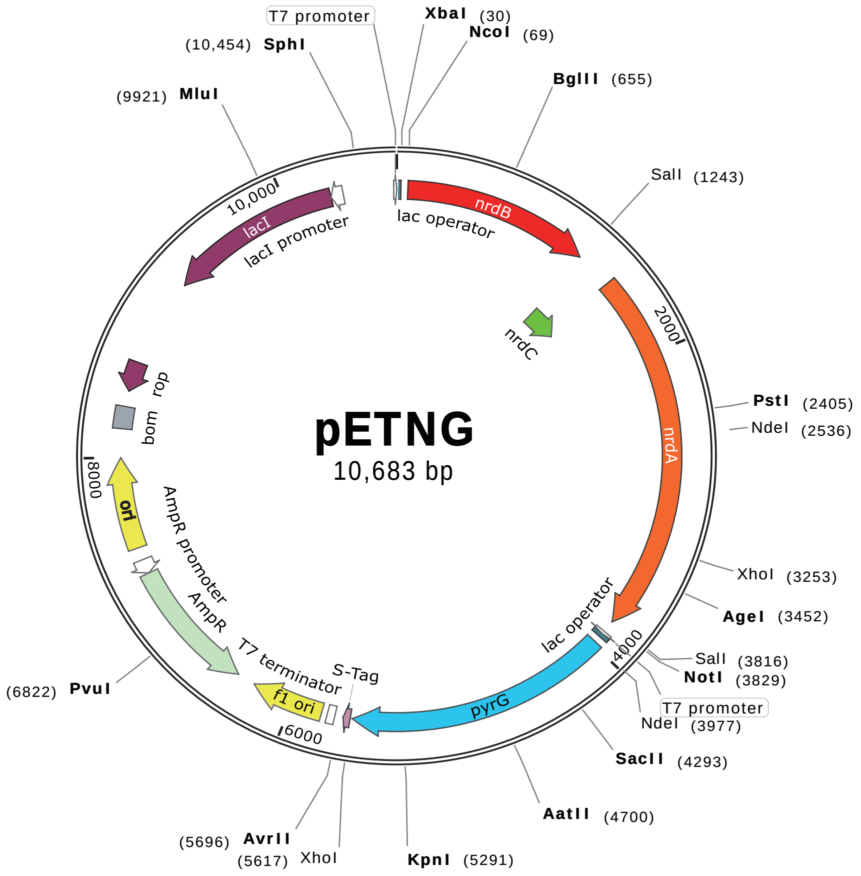


B

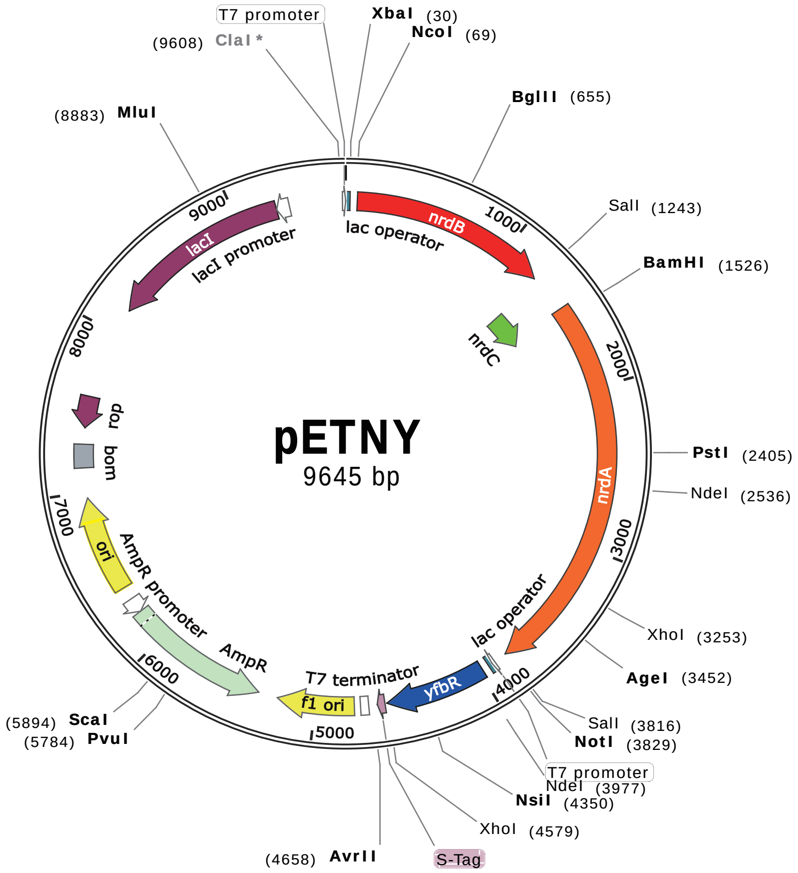


**Supplementary Figure S3.** Construction of synthetic combinatorial operon. (A) Plasmid map of pETNG. (B) Plasmid map of pETNY.

C

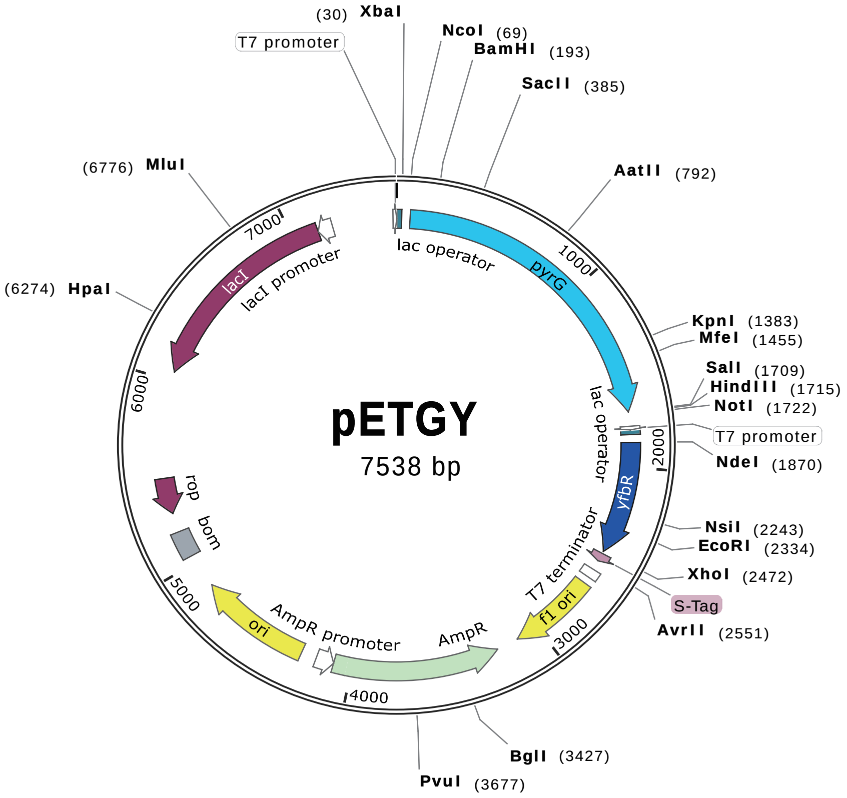


D

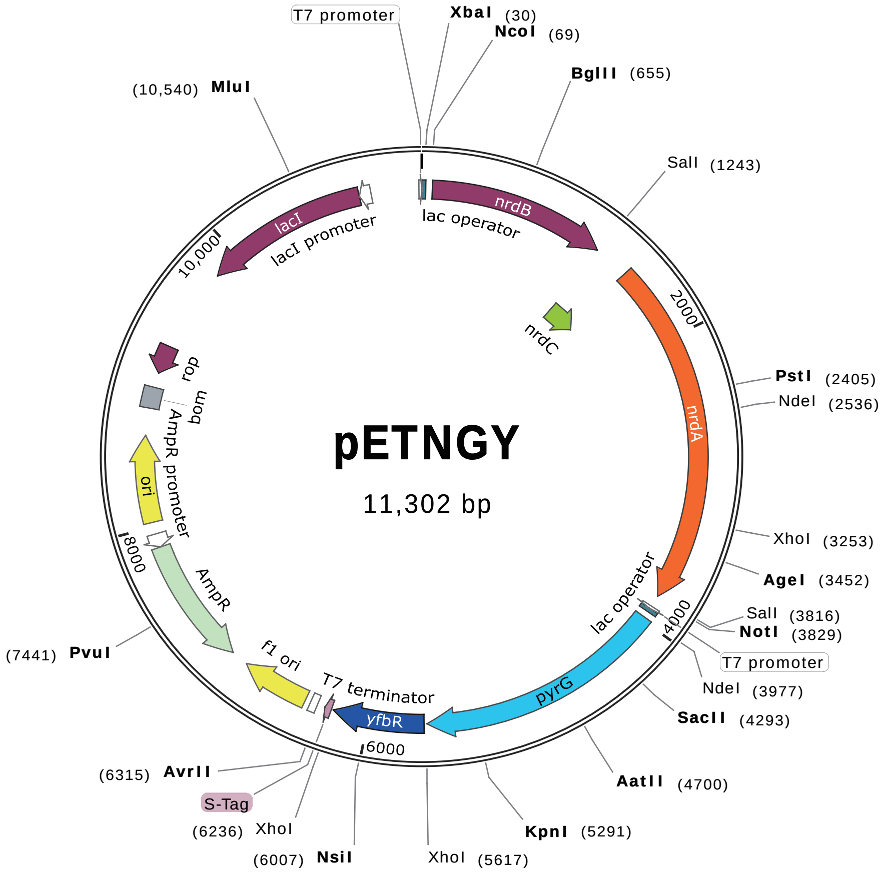


**Supplementary Figure S3**. Construction of synthetic combinatorial operon. (C) Plasmid map of pETGY. (D) Plasmid map of pETNGY.

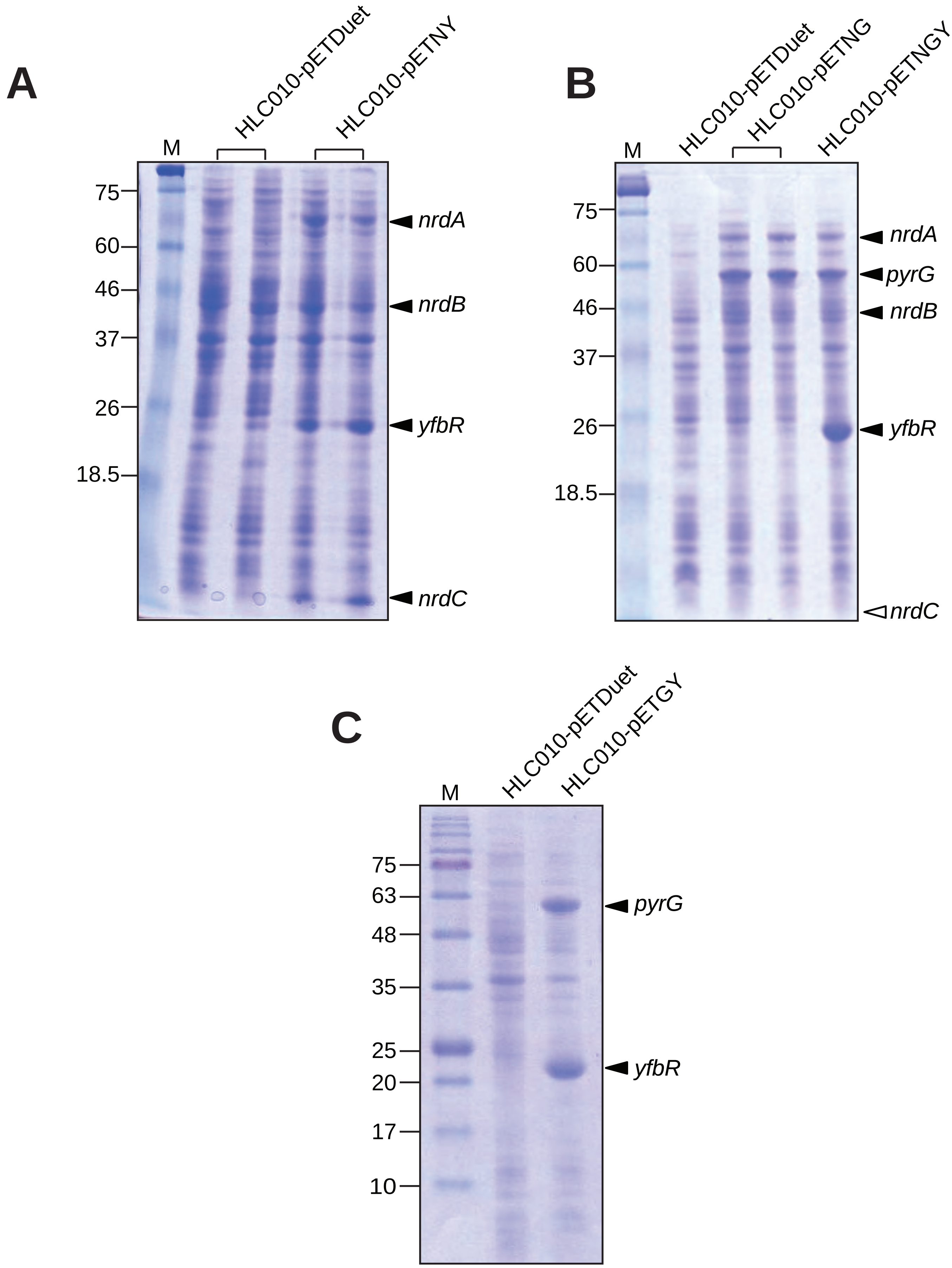


**Supplementary Figure S4**. Expression of each expression plasmid. SDS-PAGE analysis of protein expression by Coomassie staining. Molecular weight size markers are shown on the left. (A) SDS-PAGE of soluble lysate derived from HL010 cells expressing pETNY, (B) SDS-PAGE of soluble lysate derived from HL010 cells expressing pETNG and pETNGY (C) SDS-PAGE of soluble lysate derived from HL010 cells expressing pETGY.

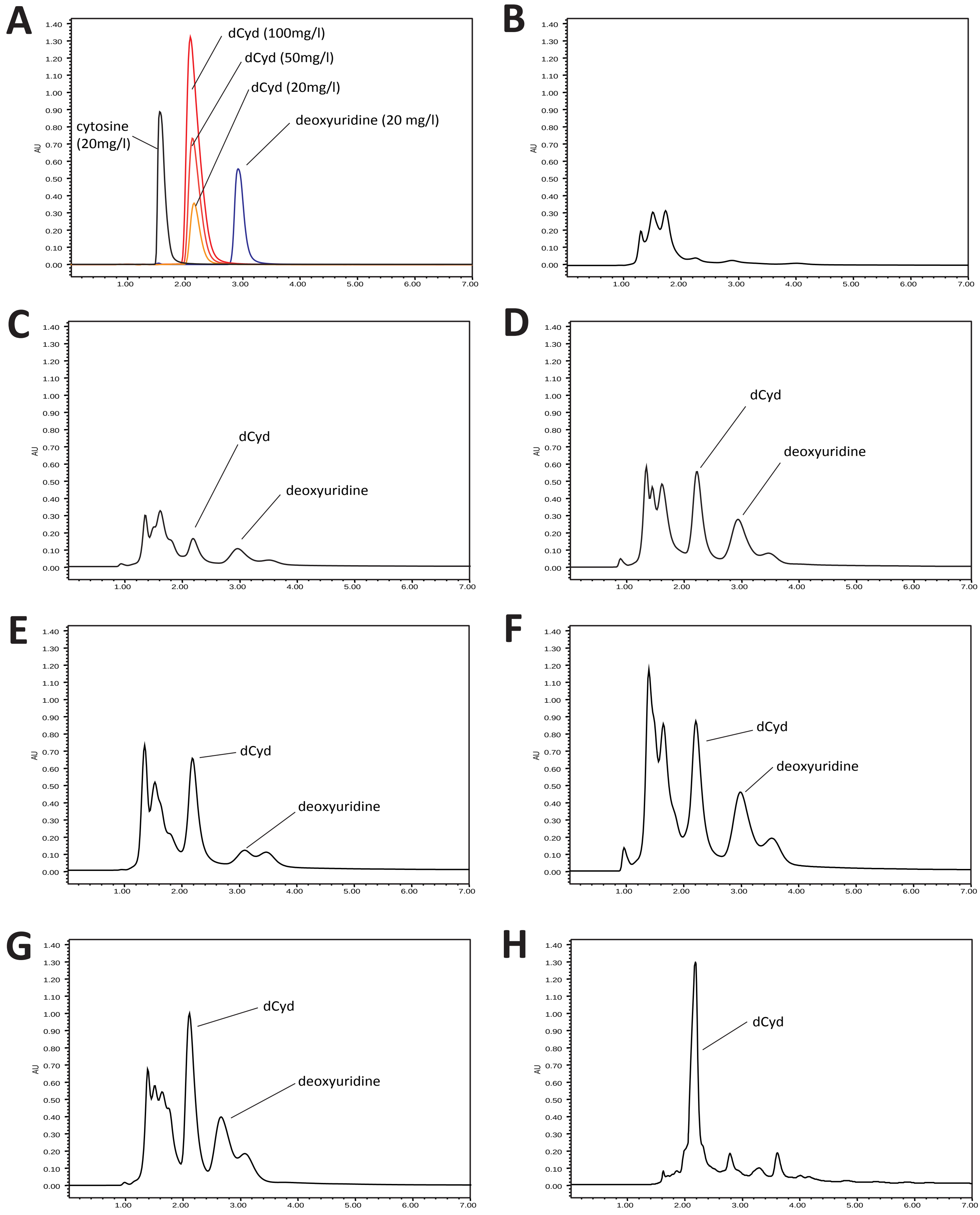


**Supplementary Figure S5.** HPLC profiles of culture supernatants. In sterile production medium, no significant nucleosides and nucleotides peaks related to this study were detected. (A) HPLC profile of standards (B) HPLC profile of supernatant prepared from HLC003. (C) HPLC profile of supernatant prepared from HLC010. (D) HPLC profile of supernatant prepared from HLC010-pETNG. (E) HPLC profile of supernatant prepared from HLC010-pETNY. (F) HPLC profile of supernatant prepared from HLC010-pETNGY. (G) HPLC profile of supernatant prepared from HLC010-pETGY. (H) HPLC profile of supernatant prepared from HLC015-pETGY.

## Supplementary Table S1. Differential expression level analysis of HLC007 and HLC010

| | SB1 | SB2 | SB3 | mean SB | SC1 | SC2 | SC3 | mean SC | Avr. Ratio | P value |
|---|---|---|---|---|---|---|---|---|---|---|
| *glnA* | 1011.1244 | 1115.7235 | 1115.7235 | 1080.8571 | 341.6903 | 557.8618 | 146.4387 | 348.6636 | 3.10 | 0.027013 |
| *gltB* | 1314.4618 | 1269.1355 | 1537.6065 | 1158.4286 | 270.4960 | 529.2313 | 82.3249 | 294.0174 | 3.94 | 0.034936 |
| *gltD* | 976.2581 | 1011.1244 | 1150.5899 | 1124.8571 | 329.9581 | 584.9257 | 209.9733 | 374.9524 | 3.00 | 0.045941 |
| *carA* | 1499.2535 | 1394.6544 | 2196.5806 | 1407.8571 | 384.4988 | 354.9220 | 147.8842 | 295.7683 | 4.76 | 0.049691 |
| *carB* | 1255.1889 | 1115.7235 | 1101.7770 | 1632.5714 | 472.0688 | 752.3597 | 250.7866 | 491.7384 | 3.32 | 0.048658 |
| *aspC* | 1398.1410 | 1436.4940 | 1453.9272 | 739.0000 | 160.4171 | 324.4390 | 55.8756 | 180.2439 | 4.10 | 0.00436 |
| *pyrB* | 209.1982 | 278.9309 | 317.2839 | 975.2857 | 1215.9406 | 1519.9258 | 1063.9481 | 1266.6048 | 0.77 | 0.019851 |
| *pyrI* | 331.2304 | 345.1770 | 348.6636 | 1066.4286 | 1055.5466 | 1414.6501 | 794.3805 | 1088.1924 | 0.98 | 0.053738 |
| *pyrC* | 313.7972 | 341.6903 | 338.2037 | 501.2857 | 490.7323 | 532.9459 | 559.3293 | 527.6692 | 0.95 | 0.004363 |
| *pyrD* | 345.1770 | 285.9041 | 268.4710 | 599.5714 | 620.4867 | 697.1761 | 773.8654 | 697.1761 | 0.86 | 0.027109 |
| *pyrE* | 296.3641 | 275.4442 | 338.2037 | 928.4286 | 971.1149 | 1387.3071 | 843.0558 | 1067.1593 | 0.87 | 0.051721 |
| *pyrF* | 477.6691 | 306.8240 | 251.0378 | 1011.4286 | 738.5034 | 882.9932 | 786.6667 | 802.7211 | 1.26 | 0.043807 |
| *argA* | 348.6636 | 310.3106 | 407.9364 | 910.4286 | 830.0966 | 981.8347 | 865.7997 | 892.5770 | 1.02 | 0.015499 |
| *argB* | 1150.5899 | 1185.4562 | 1105.2636 | 5525.8571 | 1612.4082 | 1948.3265 | 1478.0408 | 1679.5918 | 3.29 | 0.04578 |
| *argC* | 1359.7880 | 1356.3014 | 1520.1733 | 2297.8571 | 504.9612 | 737.5838 | 459.5714 | 567.3721 | 4.05 | 0.022086 |
| *argD* | 906.5253 | 1115.7235 | 1115.7235 | 1113.1429 | 326.5219 | 482.3619 | 304.2590 | 371.0476 | 3.00 | 0.010579 |
| *argE* | 306.8240 | 345.1770 | 299.8507 | 829.7143 | 857.0675 | 847.9498 | 1030.3046 | 911.7739 | 0.91 | 0.013336 |
| *argF* | 1380.7078 | 1499.2535 | 1366.7613 | 3704.4502 | 857.6806 | 912.4261 | 967.1717 | 912.4261 | 4.06 | 0.011721 |
| *argG* | 170.8452 | 230.1180 | 132.4922 | 781.4286 | 1470.9244 | 1991.8768 | 1133.8375 | 1532.2129 | 0.51 | 0.025648 |
| *argH* | 927.4452 | 976.2581 | 1202.8894 | 1039.2857 | 265.9452 | 454.9062 | 328.9322 | 349.9278 | 2.97 | 0.021629 |
| *argI* | 1314.4618 | 1366.7613 | 1366.7613 | 1020.4286 | 200.3942 | 421.8826 | 168.7530 | 263.6766 | 3.87 | 0.004669 |

**Supplementary Table S2. Oligonucleotides for gene disruption and vector construction**

| Gene | ID | Direction | Sequences |
|---|---|---|---|
| *Gene Disruption* | | | |
| *ΔdeoA* | P1 | Forward | gtttcaaaccggcgggcggcgtgcgtactgcggaagatgcgcagaaatat gtgtaggctggagctgcttc[*] |
| | P2 | Reverse | gacgggtcagatttggcagattgagcgggcctttacgaccgttatcagct catatgaatatccgcctag |
| *Δudp* | P3 | Forward | atgtccaagtctgatgtttttcatctcggcctcactaaaaacgatttacaagggctacgc gtgtaggctggagctgcttc |
| | P4 | Reverse | gaattacagcagacgacgcgccgcttccaccacgattttcaccgcatggc catatgaatatccgcctag |
| *ΔdeoD* | P5 | Forward | aaatgggcgatttcgctgacgtagttttgatgccaggcgacccgctgcgt gtgtaggctggagctgcttc |
| | P6 | Reverse | tcgcccagcagaacggattccagtgcgatttgatcatgtcgttgaaggt catatgaatatcctccttag |
| *Δdcd* | P7 | Forward | tggcttgatgccggccgtttgtcgatcaacccacgtccgccagtggagcgtattaacggc gtgtaggctggagctgcttc |
| | P8 | Reverse | cgatatttcgcatcttcacggcggttgtaaggtcgcgccgccgggccggaaagcggctc catatgaatatcctccttag |
| *Δcdd* | P9 | Forward | caacttgcggataacttgcaatctgcactggaacctattctggcagacaagtacttcccc gtgtaggctggagctgcttc |
| | P10 | Reverse | cccactgaatcaacggcgcatcggctttttctgccagaaccgcgcgctggatatccgggt catatgaatatcctccttag |
| *ΔcodA* | P11 | Forward | ataactgaaaacagcctggatgccgaacaaggtttagttataccgccgtttgtg gtgtaggctggagctgcttc |
| | P12 | Reverse | ttggcgacaaagttaataccggacattttcagcaagcggaacaggcgtga catatgaatatcctccttag |
| *ΔthyA* | P13 | Forward | atgaaacagtatttagaactgatgcaaaaagtgctcgacgaaggcacacagaaaaac gtgtaggctggagctgcttc |
| | P14 | Reverse | ttagatagccaccggcgctttaatgcccggatgcggatcgtagccttcaatctc catatgaatatcctccttag |
| *ΔpurR* | P15 | Forward | gtgatcaacaaaacacgtttcgtcgctgaagaaacgcgcaacgccgtgtggtgtaggctggagctgcttc |
| | P16 | Reverse | ttcaatggactgcggttcttcacgtttgttgacgatacgatccaacagca catatgaatatcctccttag |
| *ΔpepA* | P17 | Forward | gggtacatcagcgccctgctacgtcggggcgaactggaaggaaaaccggg gtgtaggctggagctgcttc |
| | P18 | Reverse | cccagttgtacttacgggtaaagcgtgacaggaagcaacctgcggtaatc catatgaatatcctccttag |
| *ΔargR* | P19 | Forward | ctagttaaagcatttaaagcattacttaaagaagagaaatttagctccca gtgtaggctggagctgcttc |
| | P20 | Reverse | cgaacagctctaaaatcgcttcatacaggtctttgacggtgaaaccgtta catatgaatatcctccttag |
| *Δpgi* | P21 | Forward | gcagaccgctgcctggcaggcactacagaaacacttcgatgaaatgaaag gtgtaggctggagctgcttc |
| | P22 | Reverse | cacgctttatagcggttaatcagaccattggtcgagctatcgtggctgct catatgaatatcctccttag |
| *Vector Construction* | | | |
| *nrdB* | P23 | Forward | gg ccatgg gtacagtttttaatacaaatc |
| | P24 | Reverse | gg gtcgac actccttaaaagtatttttaaaactc |
| *nrdCA* | P25 | Forward | gg gtcgac **aaggagatatacc** atgtttaaagtatatggttatgatagc[**] |
| | P26 | Reverse | gg gtcgac ttacaatttacacgctgcacaatc |
| *pyrG* | P27 | Forward | gg catatg acaacgaactatatttttgtgacc |
| | P28 | Reverse | gg ctcgag ttacttcgcctgacgtttctggaac |
| | P29 | Forward | gg ccatgg ggacaacgaactatatttttg |
| | P30 | Reverse | gg gtcgac ttacttcgcctgacgtttc |
| *yfbR* | P31 | Forward | gg catatg aaacagagccattttttgc |
| | P32 | Reverse | gg ctcgag ttacagcggt gaatcctgg |
| | P33 | Forward | gg ctcgag **aaggagatatacc** atgaaacagagccattttttgc |

[*] Underlined sequences are priming site sequences for template pKD3

[**] Bold sequences include ribosome binding site region.
